# Optimization of a diagnostic platform for oxidation–reduction potential (ORP) measurement in human plasma

**DOI:** 10.1080/13510002.2018.1456000

**Published:** 2018-03-31

**Authors:** David Polson, Nuria Villalba, Kalev Freeman

**Affiliations:** Department of Surgery, Larner College of Medicine, University of Vermont, Burlington, VT, USA

**Keywords:** Oxidation–reduction potential, oxidative stress, plasma

## Abstract

**Objectives:** Oxidation–reduction potential (ORP) measurement can demonstrate the extent of oxidative stress in patients with severe illness and/or injury. A novel ORP diagnostic platform using disposable sensors (RedoxSYS) has been validated by comparison to mass spectrometry, but the optimal methods of sample handling for best performance of the device have not been described.

**Methods:** We sought to optimize ORP measurement in human plasma under controlled conditions. We hypothesized that the anticoagulant, freeze–thawing, and storage duration would influence measured ORP levels.

**Results:** The platform was sensitive to exogenous oxidation with hydrogen peroxide and reduction with ascorbic acid. Plasma anticoagulated with heparin was more sensitive to differences in ORP than plasma prepared in citrate. ORP measurements decreased slightly after a freeze–thaw cycle, but once frozen, ORP was stable for up to one month.

**Discussion:** We confirm that ORP detects oxidative stress in plasma samples. Optimal measurement of plasma ORP requires blood collection in heparin anticoagulant tubes and immediate analysis without a freeze–thaw cycle.

## Introduction

Reactive oxygen species (ROS) are byproducts of cellular metabolism, primarily via oxidative phosphorylation, as a result of mitochondrial respiration [1,2]. In biological systems, antioxidants produced endogenously or acquired by diet detoxify these reactive intermediates to limit damage to cells [3].

Traditionally, measurements of redox states in tissue involved the use of invasive gold and platinum electrodes [4]. For clinical utility, modifications were required that would reduce improve reliability and reproducibility [4,5]. A novel platform using disposable electrodes to quantify oxidative stress in a biological sample addresses these concerns (RedoxSYS^®^; Aytu BioScience, Inc, Englewood, CO, U.S.A.). This device measures the difference between oxidants and reductants (antioxidants) or ‘oxidation-reduction potential’ (ORP), as the electrical signal produced by oxidation of an electrode under standardized conditions, providing a rapid, composite measurement of oxidative stress without determining contributions of individual molecules involved. This aggregate measurement may provide more utility than other assays that measure specific biomarkers of oxidative stress in a clinical setting. Recent studies suggest that oxidative stress arises and/or is associated with different disease states including but not limited to trauma [6,7], metabolic syndrome [8], burn [9], sepsis [6,7,8,10,11,12], and heart failure [10], following strenuous physical exercise [13,14] and male infertility [15,16]. Under these conditions, redox state can predict not only disease progression but also it can serve as a biological marker of metabolic health to monitor the pathophysiology of associated conditions.

Previous validation experiments compared comprehensive measure of plasma oxidative stress obtained from this platform to mass spectrometry analysis to confirm that specific proteins, such as human serum albumin were oxidized [7], however, no prior experiments have titrated different levels of exogenous oxidizing or reducing agents to establish the performance characteristics of the diagnostic platform in a research setting. We sought to optimize ORP measurements in human plasma under controlled conditions and establish whether freeze–thawing cycles, storage of the sample and the type of anticoagulant used to collect whole blood would influence ORP measurements.

## Materials and methods

### Human subjects

This study received approval from our Institutional Review Board (IRB), the University of Vermont Committee on Human Research in the Medical Sciences. Healthy human volunteers provided blood samples for this study. Inclusion criteria were subjects > 18 years old. Subjects were excluded if they were pregnant, or reported recent medication use, history of diabetes or other chronic medical conditions.

### Sample collection and handling

Whole blood samples were collected from healthy volunteers through a single intravenous blood draw into a syringe, and then transferred into both heparin and sodium citrate (3.2%) anticoagulant tubes and immediately centrifuged at 590.3 g, 4°C for 10 min. After centrifugation, plasma samples were either aliquoted or stored at −80°C for future use or immediately prepared and analyzed through use of the diagnostic platform.

### ORP measurements

Measurements were taken using the RedoxSYS^®^ Diagnostic System according to the manufacturer’s instructions (Aytu BioSciences). Prior to each ORP measurement, both analyzer platforms were measured for calibration; sensor chip side A was inserted into the device to ensure the device was within specification (100 ± 1 mV) and then side B was inserted to further ensure the device was working within specification (300 ± 4 mV). Following calibration, plasma samples (30 µL) were pipetted onto the exposed filter paper reservoir of a disposable electrode strip containing three electrodes: working (silver), counter (silver), and reference (silver chloride 3 M KCl) (RedoxSYS® sensor) and then inserted in a galvanostat-based reader (RedoxSYS^®^ analyzer platform). A low oxidizing current (1 nA) is applied to the sample until the charge between working and reference electrodes changes, providing an ORP reading in mV and indicating that all potentially oxidizable molecules present in the sample are oxidized. The final reading provided the aggregate measurement of the electric conductivity of plasma as the balance between oxidized and reduced molecules.

### Time course

Following centrifugation, ORP signal was measured separately from the plasma samples collected in citrate and heparin, to establish a baseline measurement. The rest of the plasma was aliquoted (100 µL) and stored at −80°C for later use. One aliquot each of citrate-derived and heparin-derived plasma was removed from the −80°C condition on different days, and placed at room temperature until they had completely thawed. The samples were analyzed at different time points 2, 4, 6, 21, and 21 days after baseline measurement, to determine the stability of sample after storage and freeze–thawing. Additionally, another set of aliquots were oxidized using 1% H_2_O_2_ (Sigma Aldrich, St. Louis, MO). The ORP signal was then measured from a citrate tube aliquot and a heparin tube aliquot to establish a time zero oxidized measurement. The rest of the aliquots were stored at −80°C for later use. As in the control time course, samples were again measured at time points 2, 4, 6, 21, and 28 days after time zero measurement to determine stability and retention of an exogenously elevated ORP signal through time and through freeze–thaw event.

### Control titrations

Both citrate and heparin plasma samples were titrated by adding increasing concentrations of 0.03, 0.1, 0.3, 1, 3, and 10% of H_2_O_2_ solutions. Additionally, another batch of plasma was titrated into 10 and 50 mM ascorbic acid solutions. Finally, one additional batch was treated by first oxidizing the plasma by adding 0.1% H_2_O_2_ prior to the addition of ascorbic acid (10 and 50 mM) in order to mimic an elevated signal of a clinical condition prior to reduction. Ascorbic acid and H_2_O_2_ stock solutions were freshly prepared prior to each experiment in distilled water.

### Statistical analysis

All ORP measurements were performed in duplicate. Data were entered into case report forms and exported to Excel for analysis. Average ORP values are reported as mean ± standard error mean (s.e.m.); *n* represents the number of replicates per experiment. All data were analyzed using two-way ANOVA statistical analyses to test for significant differences (*P* < .05). All statistical analyses and graphs were conducted through the use of GraphPad Prism6.0 (La Jolla, CA).

## Results

### Limits of detection of ORP in human plasma

**Figure 1.**
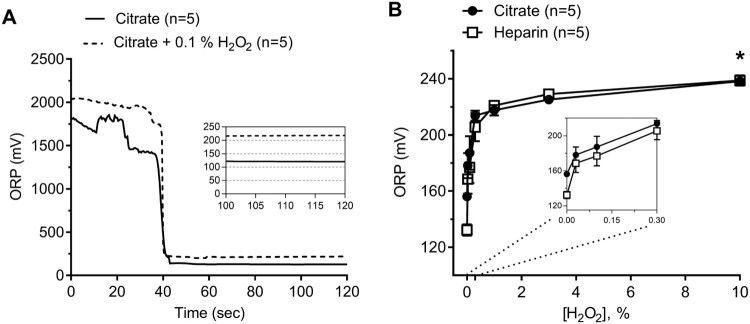
(A) Representative tracings showing oxidation–reduction potential (ORP) over time obtained from control citrated plasma in the presence and absence of 0.1% H_2_O_2_. ORP measurement is the average of the final 10 seconds. (B) Effect of the addition of known oxidant H_2_O_2_ to human plasma at incremental concentrations in both citrate and heparin anticoagulants (Two-way ANOVA; Anticoagulant *ns*; *, *P *< .05 for H_2_O_2_ vs. ORP). The inset shows data from 0–0.3% H_2_O_2_ concentrations.

We obtained fresh blood samples from 5 healthy subjects. Duplicate measurements of ORP by the same operator using two different devices showed a coefficient of variation of <2%. Figure 1(A) shows a representative tracing of citrated plasma in the presence and absence of H_2_O_2_ (0.1%) to demonstrate ORP signal perturbation by a potent oxidant agent. The addition of exogenous H_2_O_2_ to either heparinized or citrated human plasma to create different concentrations of H_2_O_2_-enriched plasma (0.03, 0.1, 0.3, 1, 3, and 10% of H₂O₂) led to increased ORP signals. ORP measurements maxed out at around 230 mV and began to plateau by a concentration of 1% H_2_O_2_ (Figure 1(B); two-way ANOVA, *P* < .0001, *n* = 5).

### Effects of anticoagulant on ORP values

Under baseline conditions, the anticoagulant affected ORP values. Plasma samples measured in heparin anticoagulant showed a marked decrease in ORP (128 ± 2.5 mV) as compared to the same samples obtained in citrate (156 ± 1.2 mV) anticoagulant (Figure 1(B); two-way ANOVA, *P* < .0001, *n* = 5). In exogenously elevated plasma, however, the type of anticoagulant had no effect on the ORP measurement at any concentration of H_2_O_2_ tested (Figure 1(B), two-way ANOVA, ns, *n* = 5).

### Effects of freeze–thawing human plasma and sample storage

**Figure 2.**
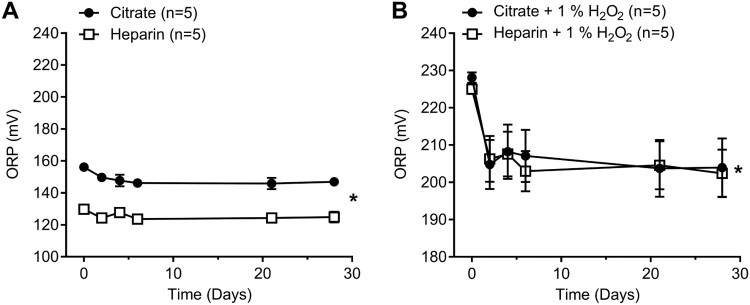
Effect of freeze–thawing human plasma in both citrate and heparin anticoagulant and stability of human plasma in storage at −80°C. (A) Effects on control human plasma up to 28 days (Two-way ANOVA; *, *P *< .05 for anticoagulant; *, *P *< .05 for time. (B) Effects on oxidized human plasma (1% H_2_O_2_) up to 28 days (Two-way ANOVA; ns, for anticoagulant; *, *P *< .05 for time.

Freeze–thawing control human plasma samples led to a decrease in ORP of 10 mV for citrated plasma and 6 mV for heparinized plasma measured at day 6 (Figure 2(A); two-way ANOVA, *P* < .05, *n* = 5). We also measured the stability of the ORP signal in oxidized samples by adding exogenous H_2_O_2_ (1%) to mimic the ORP signal obtained in a severe disease state such a sepsis or trauma [10,11,17]. Freeze–thawing exogenously elevated plasma samples with 1% H_2_O_2_ also led to a decrease in ORP signal of 25 mV and 22 mV for citrated and heparinized plasma, respectively (Figure 2(B); two-way ANOVA, *P* < .05, *n* = 5).

### Antioxidant titration on ORP measurement in human plasma

**Figure 3.**
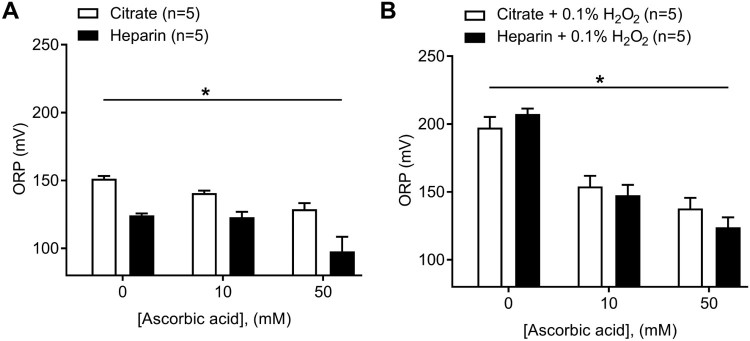
Effect of the addition of known reductant ascorbic acid to human plasma at two concentrations (10 and 50 mM) in both citrate and heparin anticoagulants. (A) Effects on control human plasma (Two-way ANOVA; *, *P *< .05 for anticoagulant; *, *P *< .05 for ascorbic acid vs. ORP). (B) Effects on oxidized human plasma (0.1% H_2_O_2_) (Two-way ANOVA; ns, for anticoagulant; *, *P *< .05 for ascorbic acid vs. ORP).

The addition of exogenous ascorbic acid at two different concentrations (10 and 50 mM) to control human plasma significantly decreased ORP signals as assessed by the diagnostic platform (Figure 3(A); two-way ANOVA, *P* < .001, *n* = 5). Control plasma measured in heparin anticoagulant also showed a greater decrease in ORP vs. citrate plasma counterparts in the presence of 10 mM ascorbic acid (9 mV drop, citrated plasma; 1 mV drop, heparinized plasma), or 50 mM ascorbic acid (22 mV drop, citrate; 26 mV, heparin) at 50 mM ascorbic acid compared to baseline values (Figure 3(A); two-way ANOVA, *P* < .0001, *n* = 5). Note that lower ORP values for heparinized plasma still persists after the addition of ascorbic acid (Figure 3(A)). The addition of exogenous ascorbic acid to plasma following the exogenous oxidation of plasma using 0.1% H_2_O_2_ also led to a decrease in ORP values of 43 and 59 mV in citrated plasma at 10 and 50 mM ascorbic acid, respectively; a 60 and 83 mV drop in heparinized plasma was obtained at 10 and 50 mM ascorbic acid, respectively, in comparison to the basal (0.1% H_2_O_2_, 0 mM ascorbic acid) measurement (Figure 3(B); two-way ANOVA, *P* < .0001, *n* = 5). We found no difference in ORP values between oxidized plasma with H_2_O_2_ prepared in heparin vs. citrate anticoagulant (Figure 3(B); two-way ANOVA, ns, *n* = 5).

## Discussion

Numerous antioxidant therapies have been proposed for treatment of shock and other medical conditions, but few methods are available to identify those individuals with an imbalance in oxidative stress most likely to benefit from these treatments [18,19,20,21]. In this study, we optimized a protocol for point-of-care estimation of oxidative stress in human plasma. We hope that future studies will utilize our protocol in the exploration of ORP as a clinical diagnostic test for oxidative stress-associated disease states including but not limited to trauma, metabolic syndrome, burns, sepsis, and heart failure.

### Effect of the anticoagulant on ORP values

We evaluated the influence of two anticoagulants (heparin and sodium citrate) on ORP values. The use of an anticoagulant is necessary because otherwise, blood samples will coagulate in the disposable test sensor, making it impossible to provide a reliable ORP measurement. We found that heparinized plasma had significantly lower baseline ORP measurements than citrated plasma. The chelation of calcium ions achieved by sodium citrate may lead to a significant increase in redox state while the potentiation of antithrombin via heparin does not have this effect. One study found that the administration of heparin during hemodialysis led to a significant decrease in the release of superoxide free radicals, which suggests an alternate explanation for why baseline heparin ORP readings were lower than citrate [22]. Interestingly, following the addition of the oxidant H_2_O_2_, ORP measurements between heparin and citrate plasma reach a steady plateau suggesting that plasma achieves a maximum oxidizable state (Figure 1(B)).

### Effect of freeze–thawing and storage of sample on ORP values

Our results suggest that there is a significant deterioration (decrease) in the ORP signal (6–10 mV drop at day 6) as a result of freeze–thaw cycles in plasma samples collected in both citrate and heparin tubes as compared to fresh plasma ORP measurements (Figure 2(A)). This drop in the ORP signal remained constant through storage at −80°C conditions of up to one month in time suggesting that, though there is an initial drop in the signal after any freeze–thaw cycle, the samples remain stable when stored at −80°C. A larger drop in ORP (22–26 mV) occurred following freeze–thawing under oxidizing conditions with H_2_O_2_ that could represent ORP values obtained under disease states. We conclude that for optimal and most accurate results, fresh ORP measurements are preferred, and fresh/freeze–thaw ORP readings are incomparable with regard to plasma.

### Positive and negative control validation of platform

The device can detect increases in ORP as a result of the exogenous addition of H_2_O_2_ (Figure 1(A,B)). Ascorbic acid was used on control plasma to determine the lower bounds of the device with regard to plasma. The ascorbic acid produced a significant reduction in the ORP signal of control plasma suggesting that plasma obtained from healthy (control) individuals also contains circulating oxidized proteins that can be further reduced. Furthermore, we confirmed that the platform can detect changes in ORP by first oxidizing control plasma with H_2_O_2_ (0.1%) and then adding ascorbic acid to decrease the ORP signal (Figure 3(B)). The data present a much more drastic decrease in ORP signal (43 and 60 mV for citrate and heparinized plasma at 10 mM ascorbic acid, respectively) in comparison to the decrease seen under basal conditions (9 and 1 mV for citrate and heparinized plasma at 10 mM ascorbic acid, respectively), as more proteins become oxidized through H_2_O_2_ treatment leading to an overall greater potential for reduction after ascorbic acid treatment. One previous study also found decreased ORP signal by ascorbate despite this study used the phosphate-buffered saline solution (PBS) rather than plasma [17]. Our data further validate the ability of the device to detect decreases in ORP signal when plasma samples are exogenously treated with an antioxidant independently of the basal ORP measurement.

In our study, ORP values obtained from healthy controls showed an average of 130 ± 2.5 mV which are in agreement with ORP values previously reported (133 ± 9 mV) under the similar conditions with heparinized plasma [17]. Published literature links disease states with increases in oxidative stress that result in an ORP signal between 150–170 mV; these ORP values are considered within a pathological range, and should lead to close patient monitoring [17].

One limitation to our study is the theoretical potential for exposure to ambient air during application of the sample to the test strip to influence the determination of ORP. However, it has been previously demonstrated that the surface of an aqueous solution provides a physical barrier for rapid oxygen equilibration due to the lower solubility and limited diffusion of gas in aqueous media. The partition of oxygen from the gas phase into the plasma would be the rate-limiting step for oxygen diffusion to the ORP diffusion to the probe is the aqueous layer diffusion to the ORP sensor. Previous measurements of the time for equilibration of 90% of [O_2_] from 25% level across a 3 µL aqueous buffer layer requires 38.4 ± 3.1 minutes [23]. Because our measurements were all performed within 2 minutes of exposure to air, the amount of oxygen expected to diffuse across the surface of the plasma samples would be minimal and should have little or no impact on ORP.

We conclude that plasma ORP measurement with the RedoxSYS system has potential utility for assessment of oxidative stress in humans. ORP measurement can detect incremental changes that either increase or decrease redox state, but optimal measurement protocols can provide improved sensitivity and help to avoid potential misinterpretations of experimental findings.
